# Chimeric Antigen Receptor T Cell Therapy for Pediatric B-ALL: Narrowing the Gap Between Early and Long-Term Outcomes

**DOI:** 10.3389/fimmu.2020.01985

**Published:** 2020-08-11

**Authors:** Liora Schultz

**Affiliations:** Division of Pediatric Hematology and Oncology, Department of Pediatrics, Stanford University, Palo Alto, CA, United States

**Keywords:** chimeric antigen receptor, B cell-malignancies, acute lymphoblastic leukemia, pediatrics, CD19 antigen, adoptive immunotherapy

## Abstract

Chimeric Antigen Receptor (CAR) T cell therapy targeting CD19 has introduced a paradigmatic shift in our treatment approach for advanced B cell malignancies. A major advance has been in the field of pediatric B-ALL where complete responses have been achieved across clinical trials with rates of 65–90% in the relapsed/refractory setting. These striking early response rates led to FDA approval of Tisagenlecleucel, CD19-specific CAR T cells, in August 2017. With broadened access and available longitudinal follow up, it is imperative to study the true durability of CAR-mediated responses and establish long-term relapse free and survival outcomes following CAR therapy. Phase I and II clinical trials have reported event-free survival rates of 50% at 1 year following CD19-CAR infusion in children and young adults with B-ALL. Here, we review some of the major challenges accounting for the discrepancy between early response rates and long term outcomes. In specific, relapse with CD19^+^ or CD19^–^ disease has emerged as a major challenge following CD19-CAR T cell therapy. Related, is the issue of CAR persistence which has been shown to correlate with long-term outcomes. We highlight select efforts to optimize clinical strategies and CAR design to promote enhanced persistence. To date, we do not have robust predictors of response durability and relapse following CAR therapy. The ability to identify patients at risk of relapse in an *a priori* manner may introduce an interventional window to consolidate CAR-mediated remissions and enhance response durability. This review highlights the need to bridge the gap between the remarkable early complete responses achieved with CD19-CAR T cell therapy and the long-term survival outcomes.

## Introduction

**FIGURE 1:**
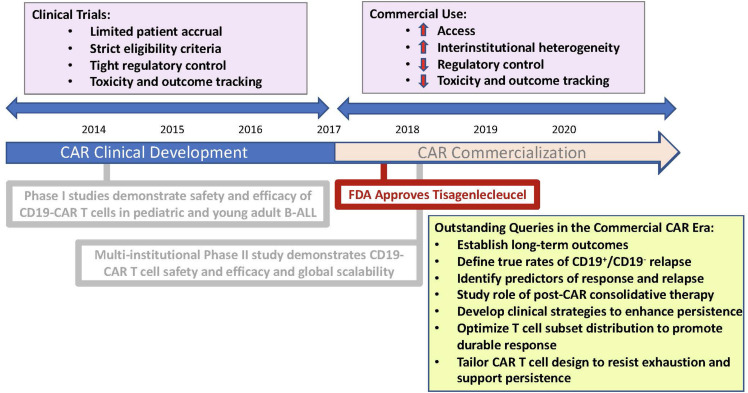
Schematic representation mapping the chronology of CAR commercialization. Illustration highlights practice changes characterizing the era of CAR commercialization and pertinent queries requiring further study.

Relapsed and refractory B cell Acute Lymphoblastic Leukemia (B-ALL) is accompanied by a dismal prognosis and accounts for a significant amount of cancer-related mortality, specifically in the pediatric population, where ALL remains the most common cancer subtype (1). The development of chimeric antigen receptor (CAR) T cell therapy has introduced a new therapeutic option for this patient population and has demonstrated remarkable clinical outcomes. In specific, CAR T cells targeting the B-cell associated antigen, CD19, has achieved complete response (CR) rates of 65–90% across clinical trials spanning institutions in patients with B-cell leukemias (2–6). These early results led to FDA approval of CD19-specific CAR T cells, Tisagennlecleucel (kymriah), in August 2017, and prompted mass efforts to permit scalability and access. The approved indication is for the treatment of pediatric and young adult patients with refractory B-ALL or B-ALL in second or greater relapse. With FDA approval, CAR T cell therapy has become available for commercial use. With broader access to CAR T cell therapies and increased experience and longitudinal follow up, it is now pertinent to understand the long-term outcomes using CAR T cell therapy (Figure 1). Here we review a limited background on CAR T cell therapy, highlight successes using CD19-CAR T cells in achieving early responses in Pediatric B-cell ALL, address the importance of identifying predictors of CAR responses and resistance and highlight factors challenging long-term CAR responses.

## Background: the CAR Construct

CARs are artificial antigen receptors engineered to fuse an antibody-binding domain targeting a tumor-specific antigen to T cell derived-signaling domain/s (7). T cells can be engineered to express CARs targeting tumor-specific antigens, endowing T cells with tumor-specific cytotoxicity. The majority of CARs designed for clinical development have targeted the B-cell associated CD19 surface antigen and have been used in B cell malignancies, including B-cell leukemias and lymphomas across ages (2–6, 8–13). To date, the majority of CARs developed for clinical trials have included a primary CD3-ζ signaling domain and a CD28 or 4-1BB secondary co-stimulatory domain, based on early pre-clinical work demonstrating independent properties of CD28 and 4-1BB in enhancing cytotoxicity beyond first-generation CARs housing a singular CD3-ζ signaling domain (7, 14, 15). Physiologic T cells rely on antigen presentation within the major histocompatibility complex (MHC) and are dependent on secondary costimulatory signals to permit effective cytotoxicity. In contrast, antibodies can bind to surface antigens in an MHC-independent manner. Tumors often downregulate MHC and can lack costimulatory ligands, rendering them immune to T-cell mediated cytotoxicity. The design of a CAR permits MHC-independent antigen-binding by the inclusion of an antibody-derived binding domain. Additionally, by including both primary T cell signaling and endogenous costimulatory signals, CARs are not reliant on tumor expression of costimulatory ligands and can effectively induce T-cell mediated cytotoxicity by inducing multiple signals with the binding of a single tumor-specific antigen (7).

## Successes Using CD19-Specific CAR T Cells in Achieving Early Complete Responses

Efficacy of CD19-specific CAR T cell therapy was first reported in small case series in adults with indolent lymphoma and chronic leukemia (8, 9, 12). Shortly after, acute B-cell lymphoblastic leukemias were found to be exquisitely sensitive to CD19-specific CAR T cell therapy (2, 6). Phase I studies in pediatric B-ALL using independent CAR constructs across institutions demonstrated conserved efficacy ranging from 65 to 90% (2–4). The challenge of scalability required organized collaboration, merging pharmaceutical and academic efforts. A phase II global, single-cohort study using Tisagenlecleucel demonstrated that the single-institution phase I outcomes were paralleled in this multi-institutional study with a CR rate of 81% following a single infusion of CAR T cells (5). This landmark study importantly demonstrated feasibility of centralized CAR manufacturing for scaled, global use and led to FDA approval of Tisagenlecleucel for children and young adults with B-ALL.

## Relapse Patterns Post-CAR and Durability of CAR-Mediated Responses

### Post-CAR Relapse

As described, CD19-CAR T cell therapy has proven effective at achieving early complete response rates. With increasing follow up, the emergence of relapse post-CAR T cell therapy, however, poses a significant clinical challenge to the durability of CAR-mediated remissions (16, 17). The two most prominent patterns of relapse include relapse with conserved CD19^+^ expression, most often in context of CAR loss, and relapse with CD19-negative or downregulated disease in context of ongoing CD19-targeted pressure. Studies using CD19-targeting CARs in adults and children with B cell malignancies across institutions demonstrate relapse rates of 30–60% with both CD19^+^ and CD19^–^ disease accounting for relapses (16). The largest phase II pediatric study described above however, identified CD19^–^ relapse to be a predominant pattern of relapse in their cohort of 75 infused patients with 15 of 16 evaluable relapses having CD19-downregulation (5). A phase I single-institution study evaluating 43 pediatric patients infused with a similar 4-1BB-based CD19-specific CAR construct demonstrated a lower rate of CD19^–^ relapse, possibly explained by decreased persistence and less ongoing targeted-pressure, with 7 of 18 relapses demonstrating CD19 negativity and 11 relapses with conserved CD19 expression (4). Mechanistic studies describe mutated CD19 RNA isoforms with alternatively spliced CD19 and underlying genetic mutations in CD19 driving truncated protein production or dysfunctional or absent transmembrane domains, that account for absence of surface CD19 in context of CD19^–^ relapse (18, 19). Studies are ongoing to identify subpopulations of B-ALL that predispose patients to CD19^–^ relapse. Patients with KMT2A (mixed lineage leukemia, MLL) rearrangement have been shown to have increased risk of CD19^–^ relapse in context of myeloid-transformation post-CAR (20, 21). To date, however, we lack extensive established predictors identifying patients at risk of relapse or at risk of specific relapse patterns. With CAR commercialization, specific relapse patterns are not captured in the form of clinical trial monitoring and tracking of CAR-mediated outcomes occurs at institutional discretion. With commercialization and extended CAR access comes a responsibility to continue to study specific relapse patterns in efforts to establish the true rate of CD19^+^ and CD19^–^ relapse in the pediatric CAR setting. Additionally, it is vital to study predictors of both CD19^+^ and CD19^–^ relapse so we can better differentiate patients likely to achieve durable responses and patients at high risk for post-CAR relapse in an *a priori* manner, permitting pre-emptive intervention to prevent relapse.

### CAR T Cell Persistence and B Cell Aplasia

Duration of B cell aplasia, as a representation of ongoing CAR T cell persistence, has been associated with durability of CAR-mediated remission. Direct testing for CAR T cell persistence can be done using PCR or flow cytometry, however, these tests remain research tests at this time and the standard for clinical CAR T cell persistence monitoring remains indirect testing of B cell apalasia. In pediatric ALL, it has been demonstrated that a longer duration of CD19-CAR persistence correlates with the durability of remission (4, 22). CD28 ad 4-1BB costimulation demonstrate distinct properties *in vivo*, with 4-1BB mediating protection against T cell exhaustion and facilitating long-term persistence and immune memory (23). Tisagenlecleucel, the CD19-specific CAR commercially approved for pediatric B-ALL, houses the 4-1BB costimulatory domain.

## Optimization Strategies to Enhance CAR T Cell Persistence

B cell and disease factors may impact CAR T cell persistence. In specific, low CD19 antigen load prior to lymphodepletion has been identified in one case series as a risk factor for early CAR T cell loss (4). A phase II study investigating the role of upfront CAR T cells in pediatric patients with refractory disease as detected by flow cytometry minimal residual disease (MRD) following consolidation (NCT03876769) is ongoing and will predominantly treat patients with low disease burden and may yield insight into the expansion and persistence potential of CAR T cells in a low disease burden setting. A novel strategy to promote *in vivo* CAR expansion uses T cells engineered to express the CD19 antigen (T antigen-presenting cells, T-APCs) as booster cells post-initial CAR infusion. Analysis of pediatric CD19-CAR recipients with low CD19-antigen burden or rapid CAR T cell loss or contraction treated with T-APCs demonstrated early evidence of secondary CAR T cell expansion, supporting *in vivo* antigen delivery as a potential approach toward reinvigorating CAR expansion and enhancing CAR persistence (24). Additionally, in efforts to augment CAR T cell persistence and reverse exhaustion, checkpoint inhibition is being clinically explored following CD19-CAR therapy, in event of incomplete-response or early CAR-T cell loss. Early data demonstrates safety of this strategy and preliminary promise, specifically in patients with early CAR-loss and bulky extramedullary disease (25).

### Selecting for T Cell Subsets

T cell factors likely contribute to CAR T cell persistence. Patients treated with CAR T cell therapy are often multiply relapsed and have had significant prior exposure to leukemia therapies including cytotoxic chemotherapies and allogeneic hematopoietic stem cell transplantation. These factors likely account for significant variation in the composition and representation of naive, memory subsets and stem central memory T cells and contribute to heterogeneity of T cell fitness across patients. Pre-clinical data demonstrate that T cell subsets of early lineage, including naive and stem central memory T cells, confer improved expansion during *ex vivo* CAR manufacturing. Specific chemotherapy agents such as cyclophosphamide and cytarabine associate with depletion of early lineage T cells in pediatric leukemia patients, supporting the impact of prior therapy on CAR T cell subset distribution and expansion potential. Culture methods including select use of IL7 and IL15 cytokines to enrich expansion of early lineage T cells are being explored (26). To date, with the exception of a single-institutional effort to evaluate CAR products of defined CD8 and CD4 T cell formulations (4, 13), the majority of clinical CAR-T cell products for B-ALL are made up of heterogeneous T cell subset distributions, reflecting the individual patient’s circulating T cell pool. Although pre-clinical data support use of naive and stem central memory T cell subsets, optimization of clinical CAR products enriched for select T cell lineage subsets to promote CAR persistence remains an area under study. It is vital to pursue measured study of T cell subset distribution of apheresis and CAR products across patients as they relate to CAR T cell persistence and CAR-mediated outcomes.

### Optimizing CAR T Cell Engineering

There is active preclinical effort to identify methods to enhance CAR signaling while mitigating CAR T cell exhaustion. CAR T cell engineering for clinical trials is primarily achieved using viral-mediated transduction with random insertion. Targeted CAR insertion to the T-cell receptor α constant (TRAC) locus using CRISPR/Cas9 has been explored as a method to avert tonic signaling and defer effector-T cell differentiation and exhaustion (27). Efforts to characterize properties driving T cell exhaustion using a tonically active CAR model identified deficiency of the AP-1 factor, c-Jun, as a driver of T cell-exhaustion. Engineering CAR-T cells to over-express c-Jun is an alternative approach to rendering CAR T cells resistant to exhaustion that has demonstrated pre-clinical promise (28).

Although, as described, costimulatory domains such as CD28 and 4-1BB are known to have variable properties driving expansion and persistence, the CAR structure is modular, housing additional transmembrane domains and immunoreceptor tyrosine-based activation motifs (ITAMs) that can be engineered to titrate function. Manipulation of quantity and position of functional ITAMs demonstrates that CD1928ζ CARs expressing a single, proximal ITAM maintain cytotoxicity while protecting against T cell differentiation and exhaustion as compared to CD1928ζ CARs with additional or distal ITAM signaling (29). Precise modulation of a 4-1BB CAR to express a CD28-hinge/transmembrane domain and inclusion of additional ITAM domains has been shown to permit CAR activity in response to lower-antigen expression while preserving 4-1BB-mediated persistence (30). Further understanding of T cell factors driving activation, persistence and exhaustion underpin our ability to engineer CAR constructs optimized for low-threshold activation and enhanced persistence.

## CAR-Mediated Toxicities

Early CAR translation identified CAR-mediated cytokine release (CRS) syndrome and immune effector cell-associated neurotoxicity syndrome (ICANs) to be the two most-common post-CAR toxicity syndromes, with CRS having increased frequency and generally preceding ICANs (11, 31–33). CRS is characterized by fever but can be associated with constitutional symptoms, vital sign instability including hypotension and hypoxia and in severe cases may warrant vasopressor-use, intubation, PICU level care and rarely result in fatalities. ICANs also spans severity with symptoms ranging from mild confusion, aphasia, impairment of cognitive or motor skills to seizures, loss of consciousness and in the rare case cerebral edema and death. Treatment for both these syndromes may be limited to supportive care or may include agents such as tocilizumab, an anti-IL-6 receptor antibody that abrogates IL-6 signaling and disrupts toxicity symptoms without compromise of efficacy, or steroids (32, 34). Initial concerns that disrupting CAR-mediated toxicity will in parallel disrupt efficacy have been dispelled and agents such as tocilizumab and steroids are currently used more liberally in response to toxicity. Additional agents are under investigation for treatment of CAR related toxicities including Siltuximab, a direct IL-6-targeting antibody, and Anakinra, an anti-IL-1 receptor antagonist that has been shown to have efficacy in CAR-mediated Hemophagocytic Lymphohistiocytosis (HLH), a post-CAR toxicity on the spectrum of severe CRS seen following CD19-CAR T cell therapy (35) and recently reported to have greater frequency following CD22-CAR T cell therapy (36). Increased disease burden has been shown to be a predictor of CAR-toxicity and studies are ongoing to establish further predictors of toxicity and understand toxicity as it relates to efficacy (11). CAR-mediated toxicities such as CRS and ICANs generally occur concurrently with CAR T cell expansion and commonly manifest within the first 28 day window post-CAR therapy. Long-term neurocognitive effects in patients experiencing neurotoxicity have yet to be established and remains an area under study. Aplasia of physiologic B cells is an additional expected on-target, off-tumor side effect of CD19-CAR T cell therapy that is effectively managed with IVIG replacement. Durability of B cell aplasia is desirable, as it represents ongoing CAR persistence (37). In the pediatric registration trial of Tisagenlecleucel, all patients responding to CAR therapy developed B cell aplasia with a probability of ongoing B cell aplasia at 6 months post-CAR of 83% (5). Related hypogammaglobulinemia has been shown to be higher in pediatric patients as compared to adult CD19-CAR recipients, likely due to decreased established antibody-producing-CD19^–^plasma cell clones in children (38). Understanding specifics of adaptive T cell immune responses and reconstitution following combined lymphodepletion and CD19-CAR T cell therapy remains an area under study.

## Role of Consolidative Hematopoietic Stem Cell Transplantation Following CAR Therapy

One additional outstanding question in the field is the role for consolidative allogeneic hematopoietic stem cell transplantation (HSCT) following CAR T cell therapy. In context of data demonstrating that despite achievement of early responses post-CAR, many patients will go on to relapse with CD19^+^ or CD19^–^ disease, HSCT has been used at many centers to consolidate CAR-mediated remissions (39). Pediatric data following use of short-lived CD19-CARs, harboring the CD28 costimulatory domain, demonstrate decreased relapse in patients consolidated with HSCT. Of 28 patients achieving minimal residual disease (MRD) negative responses by flow cytometry across pediatric CD19-CAR T cell trials at the National Cancer Institute (NCI), 2 of 21 patients consolidated with HSCT relapsed as compared to 6 relapses of 7 patients who did not undergo post-CAR HSCT (40). Long-term Phase I data using CD28-based CAR T cells in adults with ALL did not show a benefit in event-free-survival (EFS) or overall survival (OS) in patients receiving consolidative HSCT post-CAR (11), however, Phase I/II data studying 4-1BB CD19-CAR T cells in adult ALL, showed prolonged EFS in patients undergoing HSCT post-CAR (41). Data analyzing pediatric ALL patients receiving 4-1BB-harboring CD19-CAR T cells, support benefit in patients receiving consolidative HSCT post-CAR. Of 38 patients achieving remission following CD19-4-1BB CAR T cells, 3 of 13 consolidated with HSCT relapsed as compared to 20 relapses of 25 patients who did not undergo post-CAR HSCT (22). Patients who are HSCT-naive prior to CAR or who experience early CAR-loss (within 63 days) post-CAR have been identified as cohorts who may specifically benefit from consolidative HSCT (22, 42).

Due to HSCT-related toxicity risks, many centers and patients/families will opt to undergo surveillance and monitor for minimal residual disease (MRD) and B cell aplasia and only proceed with HSCT in context of loss of CAR persistence or evidence of detectable MRD. In some patients, loss of B cell aplasia will may precede disease recurrence introducing a window to proceed with HSCT while the patient is in continued remission, however, some patients will relapse concurrently with CAR loss and may lose their therapeutic window for HSCT. Additionally, many patients enter CAR after prior-HSCT, and toxicity and survival outcomes of a second HSCT are significantly inferior to outcomes following initial HSCT. To date, the decision whether to pursue a consolidative HSCT has not yet been standardized and differs across institutions and depends upon individualized factors including status of prior HSCT, CAR persistence, comorbidities, donor availability and patient/family preferences. Prospective trials on the role of first or second HSCT post-CAR remissions are essential to understanding the role of HSCT in the CAR era.

## Discussion

We have learned that CD19-specific CAR T cells are effective at achieving early remissions in relapsed/refractory pediatric and young adult B-ALL, but the rate of lasting relapse-free curative outcomes lags behind with longer follow up. A phase I intent-to-treat study of CD19-CAR T cells in pediatric and young adult patients demonstrated an event-free survival (EFS) of 50.8% at 12 months in a cohort of 45 patients. Similarly, the long-term follow up of the phase II global, multi-institutional study analyzing Tisagenlecleucel in pediatric and young adult patients demonstrated an EFS rate of 50% at 12 months in a sample size of 75 infused patients. With CAR commercialization in 2017, longitudinal data is only recently becoming available and it is imperative we understand when and if the overall and event-free survival curves flatten post-CAR T cell therapy. The promising early post-CAR responses have introduced high expectations on behalf of CD19-CAR T cell recipients. Establishing the true curative potential and limitations of CAR-T cell therapy is vital to managing clinical expectations of this therapy and identifying interventional windows to further enhance the durability of outcomes. To date, we do not have robust predictors of response durability and relapse. It is a priority to pursue further interrogation of such predictors so we can identify and differentiate patients expected to have lasting responses following CAR and patients who may achieve early remission but require further consolidative therapy to maintain responses. Additionally, extensive pre-clinical efforts are ongoing to further tailor CAR design to promote CAR-persistence and long-term immune memory.

Despite major advances with CAR clinical development and commercialization, the field remains in its infancy with many outstanding queries and areas for long-term outcome optimization. Ongoing effort is in order to narrow the gap between early response rates and long-term outcomes and further harness the power of this potent therapeutic to achieve reliable, durable CAR-mediated cures.

## Author Contributions

LS wrote the manuscript in entirety and is accountable for the content of the work.

## Conflict of Interest

The author declares that the research was conducted in the absence of any commercial or financial relationships that could be construed as a potential conflict of interest.
